# Supplementary material for: Exploring mobility patterns and social health of older Canadians living at home to inform decision aids about housing: A mixed-methods study
Source: PLoS One. 2025 Apr 17;20(4):e0320876. doi: 10.1371/journal.pone.0320876 (PMC12005502; doi:10.1371/journal.pone.0320876)
Supplement: S1 Appendix — (DOCX) [file pone.0320876.s001.docx]

| **THEME** | **Code**  **1^st^ level** | **Code**  **2^nd^ level** | **Illustrative quotations** | **Quebec (n=6)** | **Alberta (n=8)** | **Total n=14** |
| --- | --- | --- | --- | --- | --- | --- |
| **1 Physical and social assets** | | | | | | |
| Describe the range of participant's resources and factors that contribute to an individual's well-being. | 1.1 Institutional setting (place other than home) |  | *I go to different places like grocery stores, I visit all these places. That means, of course, for there, also for walking, walking. There are people in Quebec City, not just me, who go out to talk to other people.* | 4 | 6 | 10 |
|  | 1.2 Physical asset (bench, shelter) |  | *I was in the bank for well over an hour. Well, they finally got a chair so I could sit down.* | 4 | 2 | 6 |
|  | 1.3 Social asset |  |  |  |  |  |
|  |  | 1.3.1 Family-neighbor | *My daughter pops in every now and again, but I mean I never know when she's going to arrive* | 5 | 8 | 13 |
|  |  | 1.3.2 Proximity with support person | *I moved here from Vancouver, so I lived next door to my daughter and son in law yeah and two grandkids. Yeah nice. It is very nice.* | 1 | 6 | 7 |
|  |  | 1.3.3 Animal | *My life is all about dog walks. And especially the last two weeks, it's really been funny.* | 1 | 2 | 3 |
| **2 Neighborhood description** | | | | | | |
| Describes the various aspects of the participant's neighborhood. This includes urban planning, quality of the public transportation network, the necessity of using a car for commuting, and proximity to services. | 2.1 Services offer |  | *For those who don't have a car, there are gas stations and restaurants on 3rd Avenue. It's all arranged anyway. There's a pharmacy. There's a library here and another library over there.* | 6 | 7 | 13 |
|  | 2.2 Number of Stores |  | *We do a lot of shopping there simply because it's within walking distance, but if we’ve got a larger amount to do. We can't drive... we can walk up and meet, take half an hour, but it’s ok.* | 4 | 7 | 11 |
|  | 2.3 Number of parks |  | *I don't even go to the park next to me. There's not much there. There's a park at the top of the square, the park where we used to go, I say go because it's the 1st year I've fallen alone.* | 4 | 5 | 9 |
|  | 2.4 Urban environment, construction |  | *Basically, that's what's [benches] missing here, because we don't really see any benches!* | 2 | 6 | 8 |
|  | 2.5 Public transportation |  | *Because of [public] transportation and the places I can afford to go.* | 5 | 1 | 6 |
|  | 2.6 Need to use car |  | *If I like the neighborhood for... it's quiet but uh... sure not having a car I couldn't stay here I'd have to take the bus every time to get a pint of milk* | 3 | 2 | 5 |
|  | 2.7 Density |  | *With all these new duplexes we have a lot of kids. And so, you know they're all starting to get to the age where. They want to go around for Halloween.* | 3 | 1 | 4 |
| **3 Mobility description** | | | | | | |
| Describes the characteristics of participant's mobility. This includes the travel purpose, distance, weather influence, their companies, the routes taken to reach the destination, the emotions evoked by the journey and the neighborhood, and the feelings associated with the intended destination. | 3.1 Weather influence |  | *The rain stops me going out a lot because sometimes it's dangerous. Even if I have a good pair of crampons, my rain. If the wind is strong, it'll rip the umbrella to shreds. / I was home all day when it's cold I don't go out I just stay home so there we go.* | 6 | 7 | 13 |
|  | 3.2 Distance |  | *I try to walk half an hour out and half an hour back, but if if I'm hurting, which I was that day, it was a very bad walk because I could I could hardly walk. So I just I just came back home. Or I would have been out longer.* | 1 | 8 | 9 |
|  | 3.3 Take a rest during your walk |  | *The reason I take this walk is because I have many places where I can stop the walk as soon as I want to.* | 5 | 2 | 7 |
|  | 3.4 With who |  |  |  |  |  |
|  |  | 3.4.1 Family | *When I go to my doctor's appointments there. Well... it's my daughter who comes because she's too afraid I'll forget things.* | 4 | 7 | 11 |
|  |  | 3.4.2 Alone | *I walk here alone all the time. I always have my phone, but that's more in case I fall.* | 6 | 4 | 10 |
|  |  | 3.4.3 Friend | *I had a friend who volunteered with me, and she called me one day and said ¨would you like to go for a walk?¨* | 3 | 6 | 9 |
|  | 3.5 Where they go |  |  |  |  |  |
|  |  | 3.5.1 Grocery | *We go to groceries and sometimes we drive over MacLeod Trail to save on foods.* | 5 | 6 | 11 |
|  |  | 3.5.2 home-Inside exercise | *I'm always outside, I'm in my garden. I'm looking forward to March, when we'll start planting tomatoes. / We're involved with the Keep Fit Stay Fit program. Uh, that's on Monday, Wednesday, Friday from 9:30 to 10:30 and then on Saturday morning it's a, chair yoga, that we get maybe three days out of the four on the weekend.* | 4 | 6 | 10 |
|  |  | 3.5.3 Sport or leisure | *Now they're starting Monday night Bingo and card games Wednesday afternoon.* | 4 | 6 | 10 |
|  |  | 3.5.4 Stores | *I go to different places like grocery stores, Fruiterie 440, Super C, Maxi, Walmart, Rossy, I visit all these places.* | 3 | 7 | 10 |
|  |  | 3.5.5 Pharmacy | *Well, sometimes that can happen when I go to the Provigo, to the drugstore. As it is, I've set myself the goal of going to get the newspaper and my Tylenol. It's not urgent, but I try every day anyway.* | 4 | 5 | 9 |
|  |  | 3.5.6 Family or friend | *I'm waiting until spring to go to Chicoutimi to see my cousin. / Well, I'm going to my neighbor's next door, she's got a swimming pool, I'm going to see her.* | 5 | 2 | 7 |
|  |  | 3.5.7 Healthcare center | *We use the Handibus for all our medical programs.* | 1 | 5 | 6 |
|  |  | 3.5.8 Parc | *Sometimes I go to the park, 2rues away, but when the weather's nice.* | 1 | 5 | 6 |
|  |  | 3.5.9 Church | *Sometimes I come and just sit here and meditate.* | 0 | 4 | 4 |
|  | 3.6 Organization |  |  |  |  |  |
|  |  | 3.6.1 routine activity | *I went once or twice a week, perhaps. We get to walk down Heritage towards…and on Hammer Hill, towards Heritage Drive which is a city block away on the sidewalk and stop for a moment or two. And then we get to Heritage Drive and then walk back, that's about a 10-minute walk to 15 minutes.* | 3 | 5 | 8 |
|  |  | 3.6.2 Scheduled | *It's hard to find first visit Melanoma checks for cancer cells. I drove alone, new rejuvenation, new location. I switched from Southwest close to former House. Much more convenient. Scheduled a 2:30 PM. Very nice doctor and staff home at 4:50.* | 1 | 5 | 6 |
|  |  | 3.6.3 special activity | *They [niece and nephew] picked us up and they took us to the buffet to have… Was that Thanksgiving? Anyway, we went out with them to the buffet.* | 3 | 2 | 5 |
|  |  | 3.6.4 spontaneous | *I don't really know where I'm going to go depends on just something strikes me.* | 3 | 1 | 4 |
|  | 3.7 Means of transport |  |  |  |  |  |
|  |  | 3.7.1 Car | *I’m going to drive until I'm 80 and I'm just turning 78 now.* | 6 | 8 | 14 |
|  |  | 3.7.2 Walk | *I do my full walk. Going down to where you turned in when I come around and I do the loop back to my house. If I do the complete one.* | 6 | 8 | 14 |
|  |  | 3.7.3 Bus | *When you take the bus, you move from one mall to another.* | 4 | 0 | 4 |
|  |  | 3.7.4 Bike | *I got both of them [mountain bike and urban bike] / I Don't go out at night at all, but I will be because I bought myself a bike.* | 0 | 2 | 2 |
|  | 3.8 Information about the Journey/ path used |  |  |  |  |  |
|  |  | 3.8.1 The way | *There are some excellent walks in this area. Like leaving the main entrance if you walk around, there's a path down here. Oh, you can't really see it, huh? But then it goes North, and there's actually quite a little lake there.* | 6 | 7 | 13 |
|  |  | 3.8.2 Frequency | *In the summertime, we try to go out there 3 or 4 times a week.* | 5 | 6 | 11 |
|  |  | 3.8.3 Street condition (snow-ice) | *I don't walk in the wintertime a lot. But I will if it's a sunny day, if it's melting, I'll go, but with my poles I feel more comfortable, of course, but yes it does. / My study with the winter that and that's why we didn't walk and if the roads are Bad, we do not go out.* | 5 | 6 | 11 |
|  |  | 3.8.4 Meeting people during the journey | *I meet people who are into walking like me. Then we talk about the weather and our illnesses.* | 6 | 3 | 9 |
|  |  | 3.8.5 Period of the day- (morning, afternoon, evening) | *I don't go out in the evening; it doesn't interest me. Just during the day... or in the late afternoon or early evening in the summer when the heat has cooled down…* | 5 | 3 | 8 |
|  | 3.9 Feeling |  |  |  |  |  |
|  |  | 3.9.1 About the neighborhood | *I love this neighborhood. Yeah, I have nice neighbors.* | 6 | 8 | 14 |
|  |  | 3.9.2 About the journey | *It's quiet, everyone's gone to work. When I pass on rue 999 to get to rue 999, there are about 50 cars there. / The reason I take this walk is because I have many places where I can stop the walk as soon as I want to.* | 5 | 7 | 12 |
|  |  | 3.9.3 Like the place | *1st thing is that I'm proud of the city, I think it's beautiful. And at the same time, you're always discovering new things.* | 4 | 4 | 8 |
|  |  | 3.9.4 About the traffic | *I haven't been downtown for ages and I I'm getting hesitant. / There's a lot of traffic right now... and trucks and… oh it's awful.* | 2 | 3 | 5 |
|  |  | 3.9.5 About the place visit | *It's also the hospital where my mother and brother died, I have memories of them. But I don't go there for that, of course every time I go there, I have a little thought for them, but that's my goal.* | 2 | 0 | 2 |
|  |  | 3.9.6 Dislike the place | *The hill because it is going up, you have to slow down. If you walk, you won't be able to climb to the top.* | 2 | 0 | 2 |
|  | 3.10 Equipment |  |  |  |  |  |
|  |  | 3.10.1 Walking stick-cane | *I had the cane mainly because of my knee replacement.* | 2 | 4 | 6 |
|  |  | 3.10.2 Walker | *I have a walker and a cane, and I also have the two sticks, so depending on what I'm doing, I alternate with some of them.* | 1 | 4 | 5 |
|  |  | 3.10.3 Shoes stud | *The rain stops me going out a lot because sometimes it's dangerous. Even if I have a good pair of crampons... If the wind is strong, it'll rip the umbrella to shreds.* | 2 | 2 | 4 |
|  |  | 3.10.4 Telephone | *I walk here alone all the time. I always have my phone, but that's more in case I fall.* | 0 | 1 | 1 |
| **4 Approaches to mobility challenges** | | | | | | |
| Describe the actions planned and deliberate approaches aimed for the participants at improving or managing their mobility. | 4.1 Adapt |  | *What I do is I can just go down to the arcade and then take a stairway up and do it that way. It's not really handicap friendly.* | *5* | 6 | 11 |
|  | 4.2 Cancel |  | *I'm well warned (by their daughter), ¨Mom, you don't go out when it's dark¨.* | *4* | 5 | 9 |
|  | 4.3 Avoid |  | *Costco is another place that we rarely go to, mainly because there's too many people.* | *1* | 4 | 5 |
|  | 4.4 Name of the social activity |  | *We have a group called the Wednesday walkers and we had done the Wednesday walk, but then we wanted to go out of town before.* | *3* | 4 | 7 |
|  | 4.5 Continue as before |  | *I still go up the hill. So, I walk up there most days… I have there's so many places I can walk to from here* | *0* | 2 | 2 |
|  | 4.6 Choice pathway |  |  | *0* | 0 | 0 |
|  | 4.7 circulation |  |  |  |  |  |
|  |  | 4.7.1 no problem with circulation | *It (the traffic) doesn't bother me, I like there to be life, although there can be a lot of it, it wouldn't bother me, it's the opposite.* | *4* | 0 | 4 |
|  |  | 4.7.2 avoid | *(For long distances) Winter bothers me more than it used to.* | *1* | 1 | 2 |
|  |  | 4.7.3 Adapt the route |  | *0* | 0 | 0 |
|  | 4.8 Stop to go to busy place |  |  | *0* | 0 | 0 |
| **5 Navigation strategies** | | | | | | |
| Describe the strategies the participants designed to help to manage stress and overcome obstacles they encounter in their lives. | 5.1 Lost before |  |  | *0* | 0 | 0 |
|  | 5.2 Physical |  |  |  |  |  |
|  |  | 5.2.1 Point of reference | *We get to walk down Heritage towards…and on Hammer Hill, towards Heritage Drive which is a city block away on the sidewalk and stop for a moment or two. And then we get to Heritage Drive and then walk back, that's about a 10-minute walk to 15 minutes.* | *2* | 6 | 8 |
|  |  | 5.2.2 Routine activity |  | *0* | 0 | 0 |
|  | 5.3 Practice |  |  |  |  | 0 |
|  | 5.4 Social |  |  |  |  | 0 |
|  |  | 5.4.1 asked for help | *I thought I was at the right building in Sunridge there was a guy waiting outside and these buildings you know they have lots of things inside and I said to him I said, is this a total cardiology?* | *0* | 1 | 1 |
|  |  | 5.4.2 Inform before to go out |  | *0* | 0 | 0 |
|  |  | 5.4.3 With someone |  | *0* | 0 | 0 |
| **6 Impact of Covid-19 on mobility patterns** | | | | | | |
| Describes the impacts that Covid-19 had on the participants' mobility patterns. |  | 6.1 Impact from COVID-19 | *We haven't been to a restaurant. No, not for two years because of COVID.* | *0* | 5 | 5 |
| **7 Reactions to the data source** | | | | | | |
| Describes the participants' experiences with filling out the travel diary and using the GPS during the 14 days of tracking. | 7.1 Travel diary use |  | *I had a hard time with it, getting myself to follow suit, because I have a hard time associating memory with it.* | 5 | 4 | 9 |
|  | 7.2 GPS use |  | *Everyone asked me "what is it? what is it? "It's a device they gave me". Yes, that's the way the world sees it, sometimes normal, sometimes abnormal, depending.* | 6 | 6 | 12 |
