# Supplementary material for: Exploring mobility patterns and social health of older Canadians living at home to inform decision aids about housing: A mixed-methods study
Source: PLoS One. 2025 Apr 17;20(4):e0320876. doi: 10.1371/journal.pone.0320876 (PMC12005502; doi:10.1371/journal.pone.0320876)
Supplement: Attachment — Submitted filename: Response to Reviewers.docx [file pone.0320876.s003.docx]

| **Question** | **Academic Editor Comment** | **Response** | |
| --- | --- | --- | --- |
| **1** | The referee reviewed your revision and additionally raised some comments to the manuscript and recommended "Major Revision" again. I suggest the authors to follow the referee's comment and revise this manuscript carefully. | Thank you for your feedback. We appreciate the opportunity to further refine our manuscript. We carefully addressed each of the referee's points and revised the manuscript accordingly. | |
| **Question** | **Reviewer #1 Comments** | **Response** | **Page and lines** |
| **1** | 1. Definition and Scope of Key Terms “Mobility” and “housing” appear to be core terms in your study. However, the scope of these terms requires clarification. Additionally, the hypothesis mentioned on Page 5, Line 126, lacks specificity, as it only suggests that “novel insights” will be obtained. Consequently, the overall aim of the study remains unclear. Please elaborate on the definitions and scope of terms such as “mobility,” “housing,” and “housing-related decisions,” and discuss their significance to the study. Your Response: We revised the paragraph where “housing” is first introduced and provided a definition on Page 3, Lines 77–80:  “In our previous work, we found that housing decisions among older adults, i.e., the decision-making process of choosing whether to age at home or move into institutional residential care [5, 6], are the most prevalent among the difficult decisions they face [5, 7, 8].”  Reply: It is stated, “In our previous work,” but was this research conducted by the same authors? The author names differ. Additionally, what does “the most prevalent” mean in this context? Please specify the data or studies supporting this claim and clarify the connection to the referenced previous work. | First of all, thank you for giving us the opportunity to address and clarify these points. We appreciate this chance to make the manuscript clearer and more accessible for readers.  To clarify regarding the phrase "In our previous work," these studies were conducted by some members of the Canada Research Chair in Shared Decision Making and Knowledge Mobilization, the primary team responsible for Canadian data collection in this study as well. Regarding "the most prevalent," we meant the most commonly reported difficult decision for older adults, a finding that was confirmed in three studies made by this Canadian team.  However, we understood your point that the author list differs from some of the names mentioned in this article. As this article is part of a broader collaboration within a consortium that includes contributors from other countries, we have revised the paragraph as follows:  *A cross-sectional survey conducted with a pan-Canadian web-based panel of older adults aged 65 and over, assessed clinically significant decisional conflict (CSDC) [5]. The survey revealed that housing decisions—specifically, the decision-making process regarding whether to age at home or move into institutional residential care—are the most commonly reported difficult decisions faced by older adults. This finding was confirmed by studies among caregivers of older adults [6] and their home care providers [7].* | Page 3, lines 77-82. |
| **2** | We also added a second paragraph on Page 3, Lines 81–86, contextualizing the importance of examining housing decisions and older adults’ experiences:  “Although there are many conceptual frameworks regarding housing decisions [9–13], social and emotional concerns of older adults, as well as their existing physical and social assets, are often overlooked [6, 14]. As a result, a housing decision may not fully align with their preferences, increasing their distress [5]. For example, an older adult with a habit of morning walks may relocate to an area where it is difficult to walk due to busy roads.”  Reply: This paragraph highlights important context, but further elaboration is required to demonstrate how your study adds value. Please ensure the examples directly align with the study’s scope and clearly establish a gap in the literature. | Thank you for your suggestion. Below, we have revised this paragraph to highlights the gap in the literature and how our study addresses it, and adds an example directly aligned with our study:  *Studies show a strong relationship between wellbeing and social participation among older adults [8]. Other studies suggest that moving home may remove these social networks and other informal support systems [9]. One review of support for older adults making housing decisions suggests that such support is generally undermined by lack of attention to the whole person and lack of preparation for the move [10]. Secondly, while there are studies showing that mobility among older adults is important to a sense of autonomy and social engagement [11-14], few studies have explored mobility as a social asset in the context of older adults’ housing decisions [9, 15]. As an example, an older adult who is used to visiting nearby community centers may be relocated to an area with limited access to public transportation, thereby restricting their social interactions and undermining their social health. If tools that support housing decisions, such as decision aids, fail to take such considerations into account, increased decision regret is likely [16].*  We have added this reference:  16. Diori HI. A Critical Insight into Needs Assessment Technique and the Way Social Needs are Actually Assessed. Advanced Journal of Social Science. 2021;8(1):3-9. | Pages 3 and 4, lines 83-94. |
| **3** | Regarding “mobility,” we added a definition from a previous study by Meijering (2021) on Page 5, Lines 108–111:  “Mobility plays a key role in social health. While some definitions of mobility focus solely on physical ability, such as moving oneself from one place to another [24, 25], in this study, we consider that mobility also encompasses the purpose behind the movement, such as social interactions, family engagements, work, and leisure activities [26].”  Reply: Your definition of mobility includes both physical and social dimensions. However, physical mobility tracked by GPS appears to be the primary variable in your study. How do you measure these broader aspects of mobility? Will interviews supplement GPS data? Please revise the Methods section to detail how you measure and interpret “multimodal mobility.” | Thank you for your comment. We realized that our explanation of mobility was not sufficiently clear.  In our study, mobility is primarily measured by GPS, which we use to track movement and participants' travels, and complemented by daily journal entries. Our intention was to highlight that, although mobility is physical, there are factors influencing the purpose behind older adults' mobility. These influencing factors are assessed through interviews as part of our qualitative analysis.  We have revised the paragraph as follows:  *Mobility plays a key role in social health. Although the definition of mobility refers solely to the physical ability to move independently from one place to another [24, 25], there are influencing factors that shape the underlying purpose of this movement (e.g., social interactions, family engagements, work, and leisure activities) [26]. In this study, we consider mobility as a physical phenomenon, but also the influencing factors that determine its purpose.*  Regarding the methods, we described our study design as a convergent mixed-methods approach, integrating data in which one method complements the other. We have revised the paragraph explaining the combination of GPS data to measure mobility and qualitative data to capture the factors influencing movement.  *We chose this approach due to the building nature of the data [54], where the interviews deepened our understanding of the mobility captured by the GPS tracking, uncovering the factors influencing the mobility.* | Page 05, lines 116-120.  Page 10, lines 250-252. |
| **4** | We revised the hypothesis and clarified the overall goal of the study on Page 6, Lines 137–143:  “Following an earlier exploratory study [22, 41, 42], we hypothesized that mobility and social health are important dimensions in housing decisions. Therefore, with the overall goal of informing the design of decision aids, we aimed to map the activity spaces of older adults in two Canadian provinces. By combining GPS data with narrative sources (journals and interviews), we sought to highlight the importance of considering older adults’ existing social and physical assets in housing decisions.”  Reply: The hypothesis emphasizes the importance of mobility and social health in housing decisions, but this seems self-evident. What is the novel contribution of your paper? Clearly articulate what remains unknown in this area and how your study addresses these gaps. | Thanks for the comment. We appreciate the opportunity to clarify the novel contributions of our study and how it addresses existing gaps in the literature.  While the importance of mobility and social health in housing decisions may seem self-evident at a conceptual level, at a practical level, the tools that support decision making – rarely designed by older adults themselves – hardly consider these dimensions. Please see our new summary of the literature and gaps below (response to question 6).  In addition, in terms of methodology, few studies integrate objective mobility data (e.g. GPS tracking) with qualitative insights (e.g. interviews) to provide a comprehensive understanding of how older adults navigate their activity spaces and leverage their social and physical assets. Most studies focus on either social health or mobility in isolation, often only using self-reported measures that may not capture the complexity of these dimensions.  We have revised the manuscript, and the updated text now reads:  *Following earlier exploratory studies [22, 44, 45], we hypothesized that measuring the mobility patterns of older adults, in combination with qualitative interviews and journals regarding the social advantages that are associated with that mobility, would provide a portrait of their existing social and physical assets that constitutes essential information for their housing decisions. Existing research often isolates these factors [46-48] or relies on self-reported data [49-51]. Therefore, with the overall goal of informing the design of decision aids, we aimed to map the activity spaces of older adults in two Canadian provinces by combining GPS data with narrative sources (interviews).*  To support this data, we added the following references:  46. Siren A, Hakamies-Blomqvist L. Mobility and Well-being in Old Age. Topics in Geriatric Rehabilitation. 2009;25(1):3-11.  47. Morris T, Manley D, Sabel CE. Residential mobility: Towards progress in mobility health research. Prog Hum Geogr. 2018;42(1):112-33.  48. Fuller-Thomson E, Hulchanski JD, Hwang S. The housing/health relationship: what do we know? Rev Environ Health. 2000;15(1-2):109-33.  49. Rantakokko M, Portegijs E, Viljanen A, Iwarsson S, Rantanen T. Life-space mobility and quality of life in community-dwelling older people. J Am Geriatr Soc. 2013;61(10):1830-2.  50. Rosenberg DE, Huang DL, Simonovich SD, Belza B. Outdoor built environment barriers and facilitators to activity among midlife and older adults with mobility disabilities. Gerontologist. 2013;53(2):268-79.  51. Ziegler F, & Schwanen, T. ‘I like to go out to be energised by different people’: An exploratory analysis of mobility and wellbeing in later life. Ageing and Society. 2011;31(15):758-81. | Page 6, lines 147-155. |
| **5** | 3. Citations and Academic Writing On Page 5, Lines 120–122, a citation is attached to a question, which does not adhere to academic writing standards.  Your Response: We revised the paragraph to remove the question format. The updated text appears on Pages 5–6, Lines 128–134:  “For instance, it is important to identify social supports, such as friends and family, that maintain an older adult’s social health [39]. Volunteering may provide a healthy sense of self-worth, enabled by their mobility [40]. Thus, it is crucial to understand the motivations that help them remain mobile and maintain physical health. If they decide to move, we must ask whether their social and physical assets will still be available to them.”  Reply: In the sentence “If they decide to move elsewhere,” does “move” refer to relocating to a different home or neighborhood? Please clarify the intended meaning. | Thank you for your question. We have revised the sentence as you suggested:  "*if they decide to move to a different home or neighborhood…”* | Page 6, lines 142-143. |
| **6** | Conclusion Section: Even if mobility patterns are associated with social health, how do they relate to housing transitions or decisions? The causal relationship is unclear.  Your Response: We revised the Conclusion in the Abstract (Page 3, Lines 66–67):  “Mobility patterns and social assets are crucial for understanding older adults' social health and should be considered in tools designed to support housing decisions.”  Additionally, we rewrote the final sentences of the Conclusion section (Pages 34–35, Lines 770–775):  “These factors could inform housing decisions, which, in turn, could shape the design of decision aids. Incorporating these dimensions alongside conventional health-system considerations will contribute to decision-making tools that better align with older adults' needs.”  Reply: These statements largely reiterate findings from previous studies. Summarize the relationship between mobility patterns, social health, and housing transitions based on existing literature.  Return to the Introduction to establish this connection and clarify the novelty of your contribution. | Thank you for pointing out the need to summarize the relationship between mobility patterns, social health, and housing transitions.  In fact, although there is some literature on mobility and social health among older adults there is very little on this relationship in the practical context of housing decisions. We improved the summary of existing literature in the introduction; and we revised the Conclusion to highlight the novelty of our study:  *Introduction:*  *Studies show a strong relationship between wellbeing and social participation among older adults [8]. Other studies suggest that moving home may remove these social networks and other informal support systems [9]. One review of support for older adults making housing decisions suggests that such support is generally undermined by lack of attention to the whole person and lack of preparation for the move [10]. Secondly, while there are studies showing that mobility among older adults is important to a sense of autonomy and social engagement [11-14], few studies have explored mobility as a social asset in the context of older adults’ housing decisions [9, 15]. As an example, an older adult who is used to visiting nearby community centers may be relocated to an area with limited access to public transportation, thereby restricting their social interactions and undermining their social health. If tools that support housing decisions, such as decision aids, fail to take such considerations into account, increased decision regret is likely [16].*  Conclusion:  *This study underlines the importance of integrating mobility patterns and their associated social assets into housing decision frameworks. It provides guidance for the development of user-informed decision support tools, tailored specifically to Canadian older adults' housing decisions, that consider older adults’ existing physical and social assets in common real-life situations. These actionable insights can inform the creation of tools that promote healthier aging and facilitate more person-centered housing decisions.*  We revised the conclusion in the abstract section as well:  *These findings provide actionable insights to guide the development of user-informed decision support tools tailored to the housing decisions of Canadian older adults.* | Pages 3 and 4, lines 83-94.  Pages 35 and 36, lines 782-787.  Page 3, lines 65-66. |
| **7** | Minor point ・Spelling error：Neighbourhood→Neighborhood  ・Uploaded text is submitted before correction in review mode.  ・Additional comments on Introduction The following is your idea. Please revise the text by looking for findings from previous studies that support this idea.  Relevant text: While healthcare professionals and family caregivers involved in housing decisions with older adults may be conscious of their growing health burdens and safety needs, they may be less aware of these existing social and physical assets and older adults’ access to them, which should play an equally important role in the housing decision. | We reviewed the text and found that the word "neighbourhood" (the UK spelling) appears only in the titles of the referenced articles, which we have retained to ensure consistency with the original titles.  Regarding the uploaded text, we have provided two versions: a clean copy and another with tracked changes, in accordance with the journal's policy.  Regarding the additional comment on the Introduction section, we revised the text and added relevant references as recommended.  The updated text is as follows:  *While healthcare professionals and family caregivers involved in housing decisions with older adults may be conscious of their growing health burdens and safety needs [6, 7], they may be less aware of older adults’ existing social and physical assets, which should play an equally important role in the housing decision [39-41].*  We have included the following references:  6. Lognon T, Gogovor A, Plourde KV, Holyoke P, Lai C, Aubin E, et al. Predictors of Decision Regret among Caregivers of Older Canadians Receiving Home Care: A Cross-Sectional Online Survey. MDM Policy Pract. 2022;7(2):23814683221116304.  7. Claudia Lai PH, Karine V. Plourde, Lily Yeung, France Légaré. Home care providers' perceptions of shared decision-making with older clients (and their caregivers): A cross-sectional study. Nursing & health sciences. 2021;24:487-98.  39. Adekpedjou R, Stacey D, Briere N, Freitas A, Garvelink MM, Dogba MJ, et al. Engaging Caregivers in Health-Related Housing Decisions for Older Adults With Cognitive Impairment: A Cluster Randomized Trial. Gerontologist. 2020;60(5):947-57.  40. Adekpedjou R, Stacey D, Briere N, Freitas A, Garvelink MM, Turcotte S, et al. "Please listen to me": A cross-sectional study of experiences of seniors and their caregivers making housing decisions. PLoS One. 2018;13(8):e0202975.  41. Elliott J, McNeil H, Ashbourne J, Huson K, Boscart V, Stolee P. Engaging Older Adults in Health Care Decision-Making: A Realist Synthesis. Patient. 2016;9(5):383-93. | Pages 5 and 6, lines 134-137. |
