# Supplementary material for: Exploring mobility patterns and social health of older Canadians living at home to inform decision aids about housing: A mixed-methods study
Source: PLoS One. 2025 Apr 17;20(4):e0320876. doi: 10.1371/journal.pone.0320876 (PMC12005502; doi:10.1371/journal.pone.0320876)
Supplement: Attachment — Submitted filename: Response to Reviewers 20241227.docx [file pone.0320876.s004.docx]

| **Question** | **Editor** | **Response** | | |
| --- | --- | --- | --- | --- |
| **1** | The reviewer is happy with this version, but he/she further raises two minor issues. Hope that the authors can address it. | We appreciate the reviewer's positive feedback and will address the two minor issues raised to further improve the manuscript. | | |
| **Question** | **Reviewer #1** | **Previous response** | **Response** | **Page and lines** |
| **1** | **Comment 1**: Abstract Applicability: Walking interviews and in-depth interviews provide insights into physical and social assets, as well as obstacles to social health and mobility.  **Comment:** What kind of analysis did you use to analyze the qualitative and quantitative studies? | N/A. | First of all, thank you for considering our previous responses. We appreciate the opportunity to present these minor revisions.  We understand your point and we have added to the data analysis we performed for both qualitative and quantitative data to the Abstract, while keeping it within the 300-word limit.  “**Introduction**  Many tools support housing decisions for older adults but often overlook mobility patterns and social health. We explored these factors in older Canadians living at home to inform housing decisions.  **Methods**  We conducted a mixed-methods study with 20 older adults (65+) from Quebec and Alberta living independently or in senior residences with outdoor mobility. Data collection included sociodemographic information, GPS tracking, walking interviews, daily journals, and in-depth interviews. Data from interviews, which explored physical and social assets and barriers to social health and mobility, were analyzed using deductive content analysis in NVivo 12. GPS data were subjected to spatial analysis in QGIS (Quantum Geographic Information System) to map activity spaces and mobility patterns by the number and distance of activities, activity types, and modes of transportation. Daily journals were transcribed into an Excel spreadsheet and compared with GPS data. Overall analysis was guided hierarchically by qualitative data, utilizing verbatim narratives and visualization (activity space maps) to illustrate data convergence.  **Results**  Among 20 participants, 14 completed all activities, including GPS trackers. GPS maps showed participants mostly left home to drive for shopping or walking. Over 14 days, participants made an average of 10.4 (±5.8) trips and traveled 186.9 km (±130.4), averaging 16.8 km (±29.8) per day. Transportation modes included car (n=9), walking (n=5), and bus (n=2). Daily journals revealed that participants typically traveled alone. Interviews identified physical assets as libraries and supermarkets (n=10), while social assets were family support when desired (n=13) neighborhood familiarity (n=14), both contributing to social health. Winter weather was the most cited mobility barrier (n=13).  **Conclusions**  These findings provide actionable insights to guide the development of user-informed decision support tools tailored to the housing decisions of Canadian older adults.”  We have also added further details about our analysis in the body of the manuscript, as follows.  “Upon completion of data collection, interviews were transcribed, and data anonymized (manual encryption of personally identifiable information). A deductive content analysis was performed on the transcripts based on an adapted version of the Asset-Based Approach to Community Development (ABCD) framework [44, 64] using NVivo 12. Qualitative and quantitative data (socio-demographic, self-reported health and ability to perform daily tasks) were then organized into a descriptive summary. Statistical Analysis System (SAS) software was used to describe characteristics of participants.”  We have added the following reference:  64. Kretzmann JP, & McKnight, J. L. Building communities from the inside out: A path toward finding and mobilizing a community's assets. Chicago: ACTA Publications; 1993. | Pages 1 and 2, lines 39-66  Page 11, lines 262-268 |
| **2** | Comment2: Response to Question 2:  　“I see from your explanation that there is a strong relationship between wellbeing and social participation in older adults” and that ”social health may be impaired when social interaction is limited.”  So far, this seems obvious. What new value does your study offer to what these previous studies have said? | Studies show a strong relationship between wellbeing and social participation among older adults [8]. Other studies suggest that moving home may remove these social networks and other informal support systems [9]. One review of support for older adults making housing decisions suggests that such support is generally undermined by lack of attention to the whole person and lack of preparation for the move [10]. Secondly, while there are studies showing that mobility among older adults is important to a sense of autonomy and social engagement [11-14], few studies have explored mobility as a social asset in the context of older adults’ housing decisions [9, 15]. As an example, an older adult who is used to visiting nearby community centers may be relocated to an area with limited access to public transportation, thereby restricting their social interactions and undermining their social health. If tools that support housing decisions, such as decision aids, fail to take such considerations into account, increased decision regret is likely [16].  We have added this reference:  16. Diori HI. A Critical Insight into Needs Assessment Technique and the Way Social Needs are Actually Assessed. Advanced Journal of Social Science. 2021;8(1):3-9. | Thank you for your question.  We agree that our study confirms that “there is a strong relationship between wellbeing and social participation in older adults” and that “social health may be impaired when social interaction is limited,” as you say.  However, we very respectfully suggest that in our introduction we had further proposed that mobility has an important but little recognized relationship with social health, and it is this relationship, the focus of our study, that constitutes its originality. We have revised the sentences referring to mobility to highlight this:  Introduction:  “Few studies have explored *the relationship between* ***mobility and social health*** in the context of older adults’ housing decisions [9, 15] ... If tools that support housing decisions, such as decision aids, fail to take ***mobility as a social asset*** into account, increased decision regret is likely.”  We also made some small additions to the Discussion and Conclusions to remind the reader that our study is about social assets that are associated with mobility:  Discussion:  “These activities suggest a whole additional range of ***mobility-related*** social and physical assets of the older person in their current location that should be taken into account in any future design of decision aids about housing...”  Conclusion  “...despite a decline in autonomy, numerous existing social and physical assets ***associated with mobility*** make important contributions to their social health...” | Page 4, lines 90-91 and 95.  Page 33, lines 728-730  Page 35, lines 782-784 |
