# Supplementary material for: Exploring mobility patterns and social health of older Canadians living at home to inform decision aids about housing: A mixed-methods study
Source: PLoS One. 2025 Apr 17;20(4):e0320876. doi: 10.1371/journal.pone.0320876 (PMC12005502; doi:10.1371/journal.pone.0320876)
Supplement: Attachment — Submitted filename: Response to Reviewers 2020250214.docx [file pone.0320876.s005.docx]

| Reviewer #1 |  |
| --- | --- |
| Reviewer #1: Comments to the Author This research has the potential to assist older Canadians in making housing decisions; however, I would like to clarify the following points:  ・The introduction part:  1: “Mobility and housing” seem to be the core terms of your study. However, the scope of what you mean by those two terms needs to be clarified. In addition, the hypothesis from Page 5, Line 126 is not specific, as it only states the expectation that “novel insights” will be obtained. Therefore, the overall goal of the study is not clear. Please elaborate on the definition and scope of the terms “mobility,” “housing,” or “housing-related decisions” and what value they bring. | Thank you for your valuable and insightful comment.  We revised the paragraph where 'housing' is first mentioned and added a definition on page 3, lines 77-80.  *In our previous work, we found that housing decisions among older adults, i.e., the decision-making process of choosing whether they will age at home or move into institutional residential care [5, 6], are the most prevalent among the difficult decisions that they face [5, 7, 8].*  For this paragraph, we have added the following references:  6. Roy N, Dube R, Despres C, Freitas A, Legare F. Choosing between staying at home or moving: A systematic review of factors influencing housing decisions among frail older adults. PLoS One. 2018;13(1):e0189266.  8. Claudia Lai PH, Karine V. Plourde, Lily Yeung, France Légaré. Home care providers' perceptions of shared decision-making with older clients (and their caregivers): A cross-sectional study. Nursing & health sciences. 2021;24:487-98.  Additionally, we added a second paragraph on page 3, lines 81-86, to contextualize why it is important to examine housing decisions and the experiences of older adults.  *Although there are many conceptual frameworks regarding housing decisions [9-13], social and emotional concerns of older adults, as well as their existing physical and social assets, are often overlooked [6, 14]. As a result, a housing decision may not fully align with their preferences, increasing their distress [5]. For example, an older adult with a habit of morning walks may relocate to an area where it is difficult to walk due to the proximity of busy roads.*  For this excerpt, we have added the following references:  9. ES L. A theory of migration. Demography. 1966;3:47–57.  10. Lawton MP NL. Ecology and the aging process. VI ed. Washington: American Psychological Association. 619–74 p.  11. Litwak E, Longino CF, Jr. Migration patterns among the elderly: a developmental perspective. Gerontologist. 1987;27(3):266-72.  12. RF W. Why older people move: Theoretical issues. Research on Aging. 1980;2(2):141-54.  13. RM A. A behavioral model of families' use of health services. Studies CfHA, editor. Chicago: University of Chicago; 1968.  Regarding the term “mobility,” we have added a definition previously used by a team member in another article (Meijering, 2021). This modification can be found on page 5, lines 108-111.  *Mobility plays a key role in social health. While some definitions of mobility focus solely on physical ability, such as moving oneself from one place to another [24, 25], in this study, we consider that mobility also encompasses the purpose behind the movement, such as social interactions, family engagements, work, and leisure activities [26].*  The reference we have added:  26. Meijering L. Towards meaningful mobility: a research agenda for movement within and between places in later life. Ageing & Society. 2021;41(4):711-23.  Regarding the hypothesis and "novel insights," we appreciate your comment and have rewritten the overall goal of the study to make our point more directly. The updated sentence can be found on page 6, lines 137-143.  *Following an earlier exploratory study [22, 41, 42], we hypothesized that mobility and social health are importance dimensions to consider in housing decisions. Therefore, with the overall goal of informing the design of decision aids, we aimed to map a range of activity spaces of older adults in two provinces of Canada. By using GPS data combined with subjective narrative sources (journals and interviews), to highlight the importance of considering older adults’ existing social and physical assets when making housing decisions.* |
| 2: There is a lack of articles; thus, some parts are unclear what they indicate. In particular, the word “mobility” is not clear who and what kind of mobility. For example, on Page 5, Line 125: Based on these gerontological concepts, we sought a new perspective on how mobility patterns and activity spaces affect the social health of older adults. | We presume you mean articles in the grammatical sense. We have revised the paper and added articles where they are missing.  Additionally, we have added more complete definitions of terms as outlined above. |
| 3: Some of the citations need to be corrected. Especially in page 5, Lines 120-122, I have never seen a citation attached to your question. Please stick to academic writing. | We have revised this paragraph so that it is no longer phrased as a series of questions. The updated version is located on pages 5-6, lines 128-134.  *For instance, it is important to identify the social supports, such as friends and family, that currently help maintain an older adult’s social health [39]. Or volunteering in their community may provide them with a healthy sense of self-worth, and their mobility may make this possible [40]. Therefore, it is important to understand the motivations that help them remain mobile and maintain their physical health and, if they decide to move elsewhere, to ask whether these social and physical assets will still be available to them.* |
| 4: Page4, Line 99-100 　I don't know what housing transitions indicate. Even if this is some element related to housing, there is not enough evidence to strongly connect it to social health with “must therefore” in the previous sentences. | Thank you for the comment.  We have removed the reference to “housing transitions” to avoid confusion and now refer only to housing decisions, as defined above. |
| 4: Page 5, Line 132 　Instead of “suggest” at the introduction stage, try to state some findings based on the method, result, and discussion. | Thank you for the suggestion. As this sentence is related to your first question about the hypothesis, we addressed them together, revising the language to ensure the statement is more direct and concise, making it clearer and easier for readers to understand the main point. We believe these adjustments enhance the overall readability and effectiveness of the content. |
| 5: Page5, Line 136-138 Here you seem to suggest something important as the end of Introduction, but I don't know what you are stating. | As mentioned above, we have clarified the hypothesis and aims of the paper in the last paragraph of the Introduction. |
| 6: In the Sociodemographic and other self-reported information part, please describe exactly how and what variable information you obtained. For example, there is a classification of health as poor, fair, etc., but it is not clear how it was divided. | Thanks, we appreciate this comment. We added in the Sociodemographic and other self-reported information section (page 8-9, lines 194-202) the following sentence:  *For self-reported health status, we used a 5-point Likert scale [51], allowing participants to rate their health based on subjective experience, with options ranging from poor, fair, good, very good, to excellent. For questions regarding assistance required with daily tasks, we based our items on the Instrumental Activities of Daily Living (IADL) Scale, which assesses the ability to perform complex everyday tasks (e.g., managing finances and medications) evaluating whether the participants receive or provide help with those tasks [52]. Regarding technology use, we asked participants to specify the types of technology they typically use in their daily life.*  We consulted the following references:  51. Sullivan GM, Artino AR, Jr. Analyzing and interpreting data from likert-type scales. J Grad Med Educ. 2013;5(4):541-2.  52. Gold DA. An examination of instrumental activities of daily living assessment in older adults and mild cognitive impairment. J Clin Exp Neuropsychol. 2012;34(1):11-34. |
| 7: In the data analysis part, please briefly explain what kind of analysis method you used to look at the relationship between what and what. Perhaps what you want to show is that mobility patterns are associated with social health, which you mention in the conclusion. Please explain here the analytical method used to provide the evidence for this. The current description only states the purpose of combining quantitative and qualitative data and the method of data collection.  It also does not state what kind of target is the subject of the analysis. Therefore, it is not possible to determine whether the results cover the entire n=20 or just a portion of it. | Thanks for these questions.  Following the initial statement of our data analysis goals, we have included an explanation of our approach to analyzing the data, located on pages 10-11, lines 236-250.  *We conducted this analysis in a hierarchical manner, layering one source upon another, with GPS data and interviews serving as primary sources, complemented by daily journal entries. We chose this approach due to the building nature of the data [45], where the interviews deepened our understanding of the previous experiences captured by the other data sources. For instance, GPS data may reveal a participant’s choice to take a longer route to the supermarket instead of a shorter one, while interview data could indicate that this choice serves as a coping strategy to avoid a noisy avenue that causes stress. Given that we adopted a definition of mobility that considers not only the physical aspects of travel but also other factors, this iterative feedback process complemented and integrated the data sources. The combination of methods allowed us to produce a comprehensive analysis of social health in the mobility patterns of older adults [44]. For participants who did not provide all primary data sources (e.g., those who did not use the GPS properly but provided interview data), their data were still analyzed but only in the relevant sections (e.g., their responses were included in the qualitative analysis and categorized under the appropriate themes and codes).*  For your second question regarding the total sample size for the analysis, we initially intended to analyze the full sample (n=20). However, due to both external and internal factors, such as the COVID-19 pandemic and some participants not following instructions regarding collecting GPS data or not completing the daily journal, the results from each data source varied. We did address these questions in the section “Limitations of the study” (pages 33-34, lines 744-753) as follows:  *Our study has some limitations. Not all participants were able to use the GPS properly or fill out the daily journal, which limited the analysis. During the interview, some participants had negative impressions of wearing the GPS devices. Some found them uncomfortable, inconvenient, awkward (“It was amazing that my equipment did not land in the toilet”), and that the device attracted unwanted attention. One participant had privacy concerns (“it’s just too personal”), and another felt they were being tested by researchers comparing the daily journal with the GPS data. Future research assessing mobility patterns will benefit from less intrusive technology (e.g., smart watches) and improved methods for encouraging participants to complete data entries in the daily journal. In addition, the Covid-19 pandemic hampered data collection in Alberta.* |
| Result part 8: It is not clear which population (n=20, n=14, or n=7) was analyzed throughout. For example, for which population are the results for Page 15, Line326 migration? | Thank you for your valuable feedback.  As mentioned in the preceding answer, we aimed to fully analyze the entire population (n=20), but 14 agreed to use the GPS and finally 5 of them didn’t use correctly, enabling us to fully analyze data from only 9 participants. As mentioned in the methods, for a complete analysis, at least one quantitative data source (GPS) and one qualitative source (interviews) were required that would allow us to visualize the GPS data and connect it with the narratives (interviews). Since 5 participants did not use the GPS correctly, we could not converge data for those cases, resulting in a complete analysis of 9 participants and 9 activity space maps. However, all 14 participants did use the GPS over 14 days, providing valuable feedback on its usability. For this reason, we included interviews from these participants, as they enriched the analysis and offered additional insights. Regarding daily journals, we encountered the same challenges, resulting in 7 participants completing them.  Therefore, to provide clarity, we have revised each section to specifying the subset of the population analyzed in the beginning of each result section.  In the section mentioned “Mobility description: activity length, type, and means of transport” (page 17, lines 357-358) we started with the sentence:  *Of the 9 participants who provided GPS data (5 from Quebec and 4 from Alberta), we generated 9 maps describing their activity spaces.*  In the section which covers the interviews named “Physical and social assets, social and physical obstacles, adaptation strategies, and the impact of Covid-19” (page 19, lines 397-398) we started with:  *Of the 14 participants interviewed, most of the social and physical assets mentioned were related to walking.* |
| 9: Where does the discussion part start? What is the point you want to make in the discussion part? Please briefly summarize how it relates to key words such as housing decision. | The discussion starts on page 27, under the title “Discussion”. In the discussion, we first highlighted the results we considered most relevant to our objectives and then provided interpretations. We selected five topics, and mentioned how our results relate to housing decisions in each one. We have revised the conclusion of each topic to ensure its relationship to housing decisions is clear, as follows:    1) *First, activity space maps illustrated a marked difference in number and type of activities between Alberta and Quebec which may speak to both the built-environment, green-space and the physical infrastructure. In both provinces, stores were the most frequently visited places*. (page 28, lines 610-613) ... *Designers of decisions aids for* ***housing decisions*** *should take mobility into account as well as comparison of existing and future transport options, along with importance of car transportation, when they present older adults with housing options.* (page 29, lines 634-636)  2) Second, along with family and neighbourhood attachments, physical amenities such as benches, where people could rest and meet, were also important social assets ... “*As the built environment plays a significant role in influencing the mobility of older adults ... their* ***housing decisions*** *should consider factors such as the accessibility of benches in the immediate surroundings*.” (page 30, lines 668-670)  3) *Third, obstacles to social health included the weather, which was frequently expressed as an impediment to their activities outside the home* (page 31, lines 679-680) ... *decision aids about* ***housing decisions*** *for older adults in places with severe winters such as Quebec and Alberta (where temperatures of -35 degrees C are not uncommon) need to consider the impact of winter conditions*...(page 31 lines 693-696)  4) *Fourth, by combining and building on different data sources, we were able to capture various intersecting layers in the lives of our participants, providing a better understanding of their needs for information regarding upcoming* ***housing decisions*** *or for the design of decision aids* (pages 31-32, lines 698-701*) ... the development process should enable them to explain their needs and difficulties and express what they currently appreciate most about their lives ... This will ultimately empower them as co-creators of the tools that help them make their difficult* ***housing decisions*** *(page 31-32, lines 723-725).*  5) *Lastly, using multiple data collection methods and building one on top of another (46) offered a unique perspective on older adults' space and mobility patterns* (page 33, lines 726-737) .... *we explored the culture, geography and language of the two very different provinces to explore the heterogeneity of older adults’ mobility and social assets, suggesting that the future design of decision aids for* ***housing decisions*** *must be flexible to such differences*. (page 33, lines 739-742).  As mentioned, we analyzed not only the physical aspects (e.g., travel) but also the social assets. In the following paragraph, we provide a summary of our main results to remind the reader before beginning the discussion. We revised the first paragraph of the Discussion section to include keywords such as "housing decision" on page 27, lines 593-596.  *We used the concept of activity space to explore the mobility patterns and social health of Canadian older adults living at home, which could inform decisions about housing options which, in turn, could be considered in the design of decision aids for making* ***housing decisions****.* |
| Conclusion part  10: Even if mobility patterns are associated with social health, are they related to designing decisions or housing transitions? I do not understand the causal relationship. | Thank you for your question.  Our team has designed multiple decision aids, including decision aids about older adults’ housing decisions (<https://doi.org/10.1136/bmjopen-2022-061215>; <https://doi.org/10.1002/14651858.CD006732.pub4>; <https://doi.org/10.2196/53150>; <https://doi.org/10.2196/43106>). This study contributed to a more complete understanding of what older people need to consider when they plan to move, and hence will provide more useful questions and information to include in a decision aid.  As mentioned in our response to your comment above:    Although there are many conceptual frameworks regarding housing decisions [9-13], social and emotional concerns of older adults, as well as their existing physical and social assets, are often overlooked [6, 14]. Page 3, lines 81-83.  *(…)*  *For instance, it is important to identify the social supports, such as friends and family, that currently help maintain an older adult’s social health [39]. Or volunteering in their community may provide them with a healthy sense of self-worth, and their mobility may make this possible [40]. Therefore, it is important to understand the motivations that help them remain mobile and maintain their physical health and, if they decide to move elsewhere, to ask whether these social and physical assets will still be available to them.* Page 5, lines 128-134*.*  Our aim was to emphasize the value of these factors in informing decisions rather than to suggest a strict causality.  We revised our Abstract Conclusion as follows for further clarity (Page 3, lines 66-67):  “Mobility patterns and social assets are crucial for understanding older adults' social health and should be considered *in tools designed to support housing decisions*.”  To further clarify, we also rewrote the last sentences of our Conclusion section as well. Pages 34-35, lines 770-775:  *These important factors could inform decisions about housing options which, in turn, could be considered in the design of decision aids. Taking into account these often-overlooked assets, a decision aid which includes these dimensions in addition to more obvious health-system-centric considerations such as physical safety will contribute to a decision-making process that better aligns with the lived experiences of this population.* |
